# What the papers say

**DOI:** 10.1093/jhps/hnx036

**Published:** 2017-09-13

**Authors:** Ajay Malviya

The *Journal of Hip Preservation Surgery* (*JHPS*) is not the only place where work in the field of hip preservation may be published. Although our aim is to offer the best of the best, we continue to be fascinated by work that finds its way into journals other than our own. There is much to learn from it so *JHPS* has selected six recent and topical articles for those who seek a brief summary of what is taking place in our ever-fascinating world of hip preservation. What you see here are the mildly edited abstracts of the original articles, to give them what *JHPS* hopes is a more readable feel. If you are pushed for time, what follows should take you no more than 10 min to read. So here goes …

## CAN MRI SCAN ACCURATELY PREDICT THE DEGREE OF ACETABULAR CARTILAGE DAMAGE FOUND AT HIP ARTHROSCOPY?

Two studies have looked at the role of MRI scan in predicting arthroscopic findings; the first explored MRI arthrogram and the other T2 mapping and compared the findings with the intraoperative changes.

Rajeev *et al*. [1], Gateshead, UK performed a retrospective analysis of 113 patients who had MRI arthrogram and who underwent hip arthroscopy. The MRI arthrogram was performed using gadolinium injection and reported by a single radiologist. The findings were then compared with that found on arthroscopy. The sensitivity, specificity, positive predictive value (PPV), negative predictive value (NPV), accuracy and 95% CI were calculated for each pathology. For labral tear the sensitivity was 84%, specificity 64%, PPV 91%, NPV 48% and accuracy 80%; delamination a sensitivity of 7%, specificity 98%, PPV 50%, NPV 74% and accuracy 39%; chondral changes sensitivity of 25%, specificity 83%, PPV 52%, NPV 59% and accuracy 58%; femoro-acetabular impingement (CAM deformity) a sensitivity of 34%, specificity 83%, PPV 50%, NPV 71% and accuracy 66%; and synovitis a sensitivity 11%, specificity 99%, PPV 75%, NPV 77% and accuracy 77%. The study concluded that although MRI arthrogram is a useful investigation tool in detecting labral tears and helpful in the diagnosis of femoro-acetabular impingement, when it comes to the diagnosis of chondral changes, defects and cartilage delamination, the sensitivity and accuracy are low.

Morgan *et al*. [2] in a multicentred, multinational trial based in USA and Finland attempted to validate T2* mapping as an objective, non-invasive method for the prediction of acetabular cartilage damage. In a previous study, the authors established a quantitative predictive model for identifying and grading acetabular cartilage damage using a 3-T MRI with T2* mapping and this model was applied to a cohort of 27 consecutive hips. Acetabular regions of interest (ROI) were identified on magnetic resonance and graded. Each ROI was then graded in a blinded fashion by arthroscopy. Accuratesurgical location of ROIs was facilitated with a 2D map projection of the acetabulum. A total of 459 ROIs were studied. When T2* mapping and arthroscopic assessment were compared, 82% of ROIs were within 1 Beck group (of a total 6 possible) and 32% of ROIs were classified identically. Disease prediction based on receiver operating characteristic curve analysis demonstrated a sensitivity of 0.713 and a specificity of 0.804. These results validate that T2* mapping provides statistically comparable information regarding acetabular cartilage when compared to arthroscopy.

These studies demonstrate the superiority of T2* mapping over MRI arthrogram as it is quantitative, non-invasive, takes little time and does not require a contrast agent and can be used in follow-up.

## DOES PHYSIOTHERAPY IMPROVE THE OUTCOME AFTER ARTHROSCOPIC SURGERY FOR FEMOROACETABULAR IMPINGEMENT?

Bennell *et al*. [3] in a mulitcentred-study based in Australia have conducted a randomised control trial to evaluate whether formal physiotherapy (PT)-prescribed rehabilitation improves recovery compared with self-directed rehabilitation after arthroscopic surgery for femoroacetabular impingement (FAI) syndrome.

People aged ≥ 16 years with FAI syndrome scheduled for hip arthroscopy were recruited and randomly allocated to PT or control. The PT group received seven PT sessions (one pre- and six postoperative) incorporating education, manual therapy and a progressive rehabilitation programme of home, aquatic and gym exercises while the control group did not undertake PT rehabilitation. Measurements were taken at baseline (2 weeks pre-surgery) and 14 and 24 weeks post-surgery. The primary outcomes were the International Hip Outcome Tool (iHOT-33) and the sport subscale of the Hip Outcome Score (HOS) at Week 14.

Thirty participants (14 PT and 16 control) were randomized and 28 (14 PT and 14 control; 93%) and 22 (11 PT and 11 control; 73%) completed Weeks 14 and 24 measurements, respectively. For the 14-week primary outcomes, the PT group showed significantly greater improvements on the iHOT-33 (mean difference 14.2 U) and sport subscale of the HOS (13.8 U). There were no significant between-group differences at Week 24.

The study suffered from slower than expected recruitment and funding constraints and recruitment was ceased after 23 months; however, it did demonstrate that an individual PT treatment and rehabilitation programme may augment early improvements in patient-reported outcomes following arthroscopy for FAI syndrome.

## DOES LABRAL REPAIR INFLUENCE LONG-TERM OUTCOME OF HIP ARTHROSCOPY FOR FAI?

Menge *et al.* [4] from the Steadman Philippon Research Institute, Vail, CO have looked at the 10-year survivorship of hip arthroscopic intervention for FAI comparing the outcome between labral debridement and repair.

In this single-surgeon series, retrospective analyses of prospectively collected data were performed with a minimum follow-up of 10 years. The primary patient-reported outcome measure was the HOS and Activities of Daily Living (ADL) subscale. Mann–Whitney *U*-tests were used to compare outcomes between groups, and Wilcoxon signed-rank tests were used to compare preoperative with postoperative scores. Survival analysis was performed using a multivariate Cox proportional hazards model.

Seventy-nine patients who underwent labral repair and seventy-five who underwent debridement were included in the study, and 94% (145) were followed for ≥10 years. Fifty patients (34%) underwent total hip arthroplasty (THA) within 10 years following the arthroscopy. Older patients, hips with >2 mm of joint space preoperatively, and patients requiring acetabular microfracture had significantly higher prevalences of THA. The multivariate Cox proportional hazards model showed that increased age [hazard ratio (HR) for 31–51 years = 3.06, *P* < 0.001], a joint space of ≤ 2 mm (HR = 4.26, *P* < 0.001), and acetabular microfracture (HR = 2.86, *P* = 0.036) were independently associated with an increased hazard rate for THA. When the analysis was adjusted for these factors, there was no significant difference in the HR between treatment groups (HR = 1.10, *P* = 0.762). There was also no significant difference in postoperative outcome scores between groups. The debridement group demonstrated a significant increase, between the pre- and postoperative evaluations, in the HOS-ADL score (from 71 to 96; *P* < 0.001), HOS-Sport score (from 42 to 89; *P* < 0.001), modified Harris hip score (mHHS) (from 62 to 90; *P* < 0.001), and Short Form-12 physical component summary (SF-12 PCS) score (from 43 to 56; *P* < 0.001). The repair group also demonstrated a significant increase in the HOS-ADL score (from 71 to 96; *P* < 0.001), HOS-Sport score (from 47 to 87; *P* < 0.001), mHHS score (from 65 to 85; *P* < 0.001), and SF-12 PCS score (from 41 to 56; *P* < 0.001).The median patient satisfaction score was 10 (very satisfied) in both groups.

The authors concluded that hip arthroscopy for FAI with labral debridement or repair resulted in significant improvements in the patient-reported outcomes and satisfaction without demonstrating any significant difference between the two groups. Higher rates of conversion to THA were seen in older patients, patients treated with acetabular microfracture, and hips with ≤ 2 mm of joint space preoperatively, regardless of labral treatment.

## WHAT FACTORS PREDICT CONVERSION TO THA AFTER ARTHROSCOPY?

Redmond *et al*. [5] have proposed a risk calculator using an algorithm that can predict the likelihood that a patient who undergoes arthroscopic hip surgery will undergo THA within 2 years.

The study group included patients undergoing hip arthroscopy for a labral tear, who eventually had conversion surgery to THA and this was compared with a control group of patients who underwent hip arthroscopy for a labral tear but who did not undergo conversion surgery to THA during the same study period.

Of the 893 who underwent surgery during that time, 792 (88.7%) were available for follow-up at a minimum of two years. Multivariate regression analyses of 41 pre- and intraoperative variables were performed. Variables simultaneously associated with conversion to THA in this model were older age (rate ratio RR, 1.06; *P* < 0.0001), lower preoperative mHHS (RR, 0.98; *P* = 0.0003), decreased femoral anteversion (RR, 0.97; *P* = 0.0111), revision surgery (RR, 2.4; *P* = 0.0193), femoral Outerbridge Grades II–IV (Grade II: RR, 2.23, *P* = 0.023; Grade III: RR, 2.17, *P* = 0.024; Grade IV: RR, 2.96, *P* = 0.007), performance of acetabuloplasty (RR, 1.83; *P* = 0.038), and lack of performance of femoral osteoplasty (RR, 0.62; *P* = 0.081).

Using the results of the multivariate regression the authors propose the use of a simplified calculator they have developed that may be helpful in counseling a patient regarding the risk of conversion to THA after hip arthroscopy.

## A TRAFFIC LIGHT GRADING SYSTEM OF HIP DYSPLASIA TO PREDICT THE SUCCESS OF ARTHROSCOPIC HIP SURGERY

Grammatopoulos *et al.* [6] from Reading, UK set out to determine the 7-year joint preservation rate after hip arthroscopic surgery in hip dysplasia and identify anatomic and intraoperative features that predict the success of hip preservation with arthroscopic surgery, allowing the formulation of an evidence-based classification system.

Between 2008 and 2013, 111 hips with dysplastic features [acetabular index (AI) > 10° and/or lateral center-edge angle (LCEA) <25°] that underwent arthroscopic surgery were identified. Clinical, radiological, and operative findings and the type of procedure performed were reviewed. Radiographic evaluations of the operated hip (AI, LCEA, extrusion index) were performed. Outcome measures included whether the hip was preserved (i.e. did not require arthroplasty) at follow-up and the pre- and postoperative Non-Arthritic Hip Score (NAHS) and Hip disability and Osteoarthritis Outcome Score (HOOS). The AI and LCEA were calculated, factored by a measure of articular wear (AIf and LCEAf, respectively), according to the University College Hospital, London (UCL) grading system as follows: AIf = AI × (number of UCL wear zones + 1), and LCEAf = LCEA/(number of UCL wear zones + 1). A contour plot of the resulting probability value of failure for every combination of AIf and LCEAf allowed for the determination of the zones with the lowest and highest incidences of failure to preserve the hip.

The mean AI and LCEA were 9.8° and 18.0°, respectively. At a mean follow-up of 4.5 years (range, 0.4–8.3 years), 33 hips had failed, requiring hip arthroplasty. The 7-year joint survival rate was 68%. The mean improvements in the NAHS and HOOS were 11 (*P* = 0.001) and 22.8 (*P* < 0.001) points, respectively. The zone with the greatest chance of joint preservation (odds ratio, 10; *P* < 0.001) was the green zone, with an AIf of 0°–15° and an LCEAf of 15°–25°; in contrast, the zone with the greatest chance of failure (odds ratio, 10; *P* < 0.001) was the red zone, with an AIf of 20°–100° and an LCEAf of 0°–10°.

They concluded that the 7-year hip survival rate in hip dysplasia appears inferior compared with that reported in FAI (78%). Hip arthroscopic surgery is associated with an excellent chance of hip preservation in mild dysplasia (green zone: AI = 0°–15°, LCEA = 15°–considering arthroscopic options in cases of severe dysplasia (red zone: AI > 20° and/or LCEA < 10°).

## WOULD PARTICIPATION IN SPORTS HAVE A NEGATIVE BEARING ON THE RESULTS OF PERIACETABULAR OSTEOTOMY?

Hara *et al*. [7] from Kyushu University, Fukuoka, Japan have investigated the impact of participation in sports on the progression of osteoarthritis after periacetabular osteotomy (PAO). This retrospective case-control study includes 161 patients (183 hips) who underwent PAO for symptomatic acetabular dysplasia with preoperative Kellgren–Lawrence (KL) Grade 1 or 2 between 1998 and 2011. The mean age at the time of surgery was 42.0 years (range, 12–64), and the mean follow-up duration was 100 months (range, 13–180). Data included participation in sports, the University of California, Los Angeles (UCLA) activity scale score, age at the time of surgery, body mass index, follow-up duration, history of treatment for developmental hip dislocations, Merle d’Aubigné-Postel score, Oxford Hip Score, center-edge angle, and KL grade. Univariate and multivariate analyses were applied to determine which factors were associated with progression to KL Grade 3 or 4 after PAO.

The number of patients who participated in sports significantly increased from 50 (31.1%) pre- to 89 (55.3%) postoperatively. The mean UCLA score significantly increased from 4.7 ± 2.1 pre- to 5.5 ± 2.0 postoperatively. The KL grade progressed to Grade 3 or 4 in 16 hips, including 4 hips that underwent conversion to THA. No significant differences were found in postoperative participation in sports 89 hips (53.3%) versus 11 hips (68.8%), respectively; *P* = 0.24) and the UCLA score (5.6 versus 5.1, respectively; *P* = 0.30) between hips with KL Grade 1 or 2 and KL Grade 3 or 4. A multivariate analysis revealed that no factors, including postoperative participation in sports, were significantly associated with progression to KL Grade 3 or 4.

The authors concluded that postoperative participation in sports after PAO did not significantly and negatively influence progression of the KL grade at midterm follow-up.

## CONFLICT OF INTEREST STATEMENT

None declared.
